# Neuropsychiatric Systemic Lupus Erythematosus: A 2021 Update on Diagnosis, Management, and Current Challenges

**DOI:** 10.7759/cureus.17969

**Published:** 2021-09-14

**Authors:** Sobia Sarwar, Alaa S Mohamed, Sylvette Rogers, Shah T Sarmast, Saurabh Kataria, Khalid H Mohamed, Muhammad Zain Khalid, Mohammad Omar Saeeduddin, Saher T Shiza, Sarfaraz Ahmad, Anum Awais, Romil Singh

**Affiliations:** 1 Neurology, Independent Medical College, Faisalabad, PAK; 2 Neurology, Augusta University, Augusta, USA; 3 Family Medicine, Caribbean Medical University, Des Plaines, USA; 4 Neurology, California Institute of Behavioral Neurosciences and Psychology, Fairfield, USA; 5 Neurology, Ochsner Louisiana State University Health Sciences Center - Shreveport, Shreveport, USA; 6 Neurology and Neurocritical Care, University of Missouri Health Care, Columbia, USA; 7 Neurology, West Virginia University, Morgantown, USA; 8 Anatomical Sciences, St. George's University - School of Medicine, St. George’s, GRD; 9 Internal Medicine, Liaquat National Hospital and Medical College, Karachi, PAK; 10 Psychiatry, Liaquat National Hospital and Medical College, Karachi, PAK; 11 Internal Medicine, Deccan College of Medical Sciences, Hyderabad, IND; 12 Internal Medicine, Saint James School of Medicine, Chicago, USA; 13 Internal Medicine, Fatima Jinnah Medical University, Lahore, PAK; 14 Critical Care, Mayo Clinic, Rochester, USA

**Keywords:** neuropsychiatric sle, cns lupus, clinical presentation, pathogenesis, diagnosis, management

## Abstract

Patients with systemic lupus erythematosus (SLE) experience neuropsychiatric symptoms. The term neuropsychiatric SLE (NPSLE) is a generic term that refers to a series of neurological and psychiatric symptoms directly related to SLE. In approximately 30% of patients with neuropsychiatric symptoms, SLE is the primary cause (NPSLE), and symptoms manifest more frequently around SLE onset. Neurovascular and psychotic conditions can also lead to NPSLE. Pathogenesis of NPSLE is implicated in both neuroinflammatory and ischemic mechanisms, and it is associated with high morbidity and mortality. After diagnosing and assigning causality, NPSLE treatment is individualized according to the type of neuropsychiatric manifestations, type of the predominant pathway, activity of SLE, and severity of the clinical manifestations. There are many problems to be addressed with regards to the diagnosis and management of NPSLE. Controlled clinical trials provide limited guidance for management, and observational cohort studies support symptomatic, antithrombotic, and immunosuppressive agents. The purpose of this review was to provide a detailed and critical review of the literature on the pathophysiology, diagnosis, and treatment of NPSLE. This study aimed to identify the shortcoming in diagnostic biomarkers, novel therapies against NPSLE, and additional research needs.

## Introduction and background

Systemic lupus erythematosus (SLE) is a multisystem autoimmune disease characterized by the involvement of almost every organ of the body, a broad spectrum of clinical manifestations, and several immune-mediated abnormalities leading to multiple organ dysfunction [1]. The interplay in disease development and progression results from genetic predilection, hormonal factors, and environmental triggers, leading to heterogeneous clinical manifestation, indicating the complex montage of disrupted molecular pathways in SLE. SLE generally affects females of childbearing age range from 15 to 44 years with a ratio of 13:1 in females compared to males [2]. Center for Disease Control and Prevention reported almost 0.32 million cases of SLE in the USA in 2021 [3]. Prevalence of SLE is rising, most likely due to an increase in early diagnosis of the disease and improved survival with advancements in disease pathology, diagnosis, and treatment. SLE incidence almost tripled in the last 40 years. However, the mortality rate is still higher and reported to be three times higher as compared to the healthy individuals, and mortality rate increases as the disease progress and is associated with disease-associated risk factors, pulmonary or hematological disorders, nephropathy, association with antiphospholipid syndrome, or presence of neuropsychiatric complications [4].

SLE also affects the nervous system among the broad spectrum of clinical manifestations, causing various manifestations of the central nervous system (CNS) and peripheral nervous system (PNS). Neuropsychiatric SLE (NPSLE) is a severe complication characterized by neurological and psychiatric manifestations of SLE [5]. Manifestations of NPSLE range from localized or isolated to diffuse, peripheral, and/or CNS and from mild to severe [6,7]. Diagnosis of NPSLE can be challenging for rheumatologists due to the lack of specific and sensitive laboratory serum or CSF biomarkers, radiological imaging changes, other formal criteria in establishing the diagnosis, and guiding the treatment and management decisions in NPSLE [5]. In this review article, we have provided a recent update on the diagnosis and management of the NPSLE, future directions, and the challenges.

## Review

Neuropsychiatric SLE

NPSLE refers to multiple neuropsychiatric manifestations directly related to SLE [8]. NPSLE is different from other aspects of SLE due to its development without serological changes. Prevalence of NPSLE is reported in many epidemiological studies and has suggested differences in both NPSLE and SLE, based on age, sex, and ethnicity. There is a greater incidence of neurological manifestations in females, and seizure risk is reported higher in males than females [9]. NPSLE is more frequently reported in African descendants and Asians as compared to white individuals; however, the severity of NPSLE is reported more in White patients [10,11]. Neuropsychiatric manifestations occur in the early stages of SLE and represent 39%-50% of SLE patients [12]. A meta-analysis reported the prevalence of NPSLE in 5,057 patients and underlined prevalence of 44.5% in prospective studies and 17.6% in retrospective studies. This study also included minor and nonspecific symptoms such as mild depression and anxiety. After excluding these minor symptoms, the reported prevalence was 4.3%, and incidence was 7.8% [13]. Another study reported a 12.4% prevalence of NPSLE among 308 patients diagnosed with SLE. The reported prevalence of NPSLE varies from 6% to 91%, and this variability is due to research method variances such as screening methodology, study design, follow-up duration, heterogenous measures, and lack of research method specificity. NPSLE is a severe complication of SLE, affecting the quality of life with increased morbidity and mortality [14].

Clinical manifestations of NPSLE

NPSLE can be focal or diffuse, and clinical manifestations may range from subtle cognitive dysfunction to acute confusional states, seizure disorders, and psychosis. However, headaches, anxiety, mood, and cognitive disorders are the most frequent neuropsychiatric manifestations of SLE. Cerebrovascular disease, neuropathies, acute confusional states, and seizure disorders are the most frequent manifestations associated with NPSLE, suggesting several pathogenetic mechanisms in NPSLE similar to our current understanding of the extracranial manifestations of SLE [6,15,16].

The American College of Rheumatology (ACR) published a consensus statement that defined 19 NP syndromes. These NP syndromes can be divided into 12 CNS and seven PNS syndromes, and additionally, these were classified into focal neurological syndromes and diffuse neuropsychological syndromes (Table 1) [17]. Among these syndromes, some are more frequent (6.4%-8%), and the remaining are frequent (7%-20%), infrequent (0.6%-11%), or rare (0.08%-2%) [18]. NP syndromes such as chronic inflammatory demyelinating polyneuropathy, neuromyelitis optica spectrum disorder, and small fiber neuropathy also occur in SLE; however, these are not included in this classification. This classification is not based on any clear physiological and pathological mechanism: however, it supports diagnosing SLE in the setting of neurological involvement [19].

**Table 1:** Clinical syndromes in neuropsychiatric SLE NR: Not reported, GBS: Guillian Barre syndrome.

Syndromes	Central Nervous System		Frequency (%)	Peripheral Nervous System	Frequency (%)
Neurological syndromes	Focal	Seizure disorder	7.0-20	Autonomic disorders	0.08-1.3
		Aseptic meningitis	0.3-2.7	Myasthenia gravis	0.2
		Demyelinating syndromes	0.9-2.7	Polyneuropathy	1.5-5.4
		Myelopathy	0.9-3.9	Cranial neuropathy	1.0
		Headache	12.2-28.3	GBS	0.08-1.2
		Cerebrovascular disease	8.0-15	Mononeuropathy	0.9-6.9
		Movement disorders	0.9	Plexopathy	NR
Neuropsychiatric syndromes	Diffuse	Anxiety disorders	6.4-40		
		Psychosis	0.6-11		
		Acute confusional state	0.9-7		
		Cognitive dysfunction	6.6-80		
		Mood disorders	7.4-65		

Proposed pathogenesis

**Figure 1:**
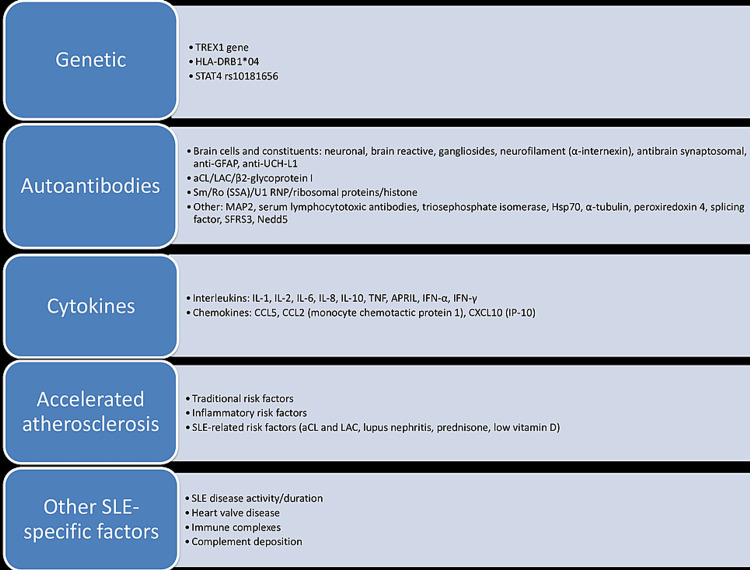
Factors involved in the pathogenesis of neuropsychiatric SLE TNF: tumor necrosis factor, HLA: human leukocyte antigen, IL: interleukin, GABA: gamma-aminobutyric acid, UCH-L1: ubiquitin carboxyl-terminal hydrolase isozyme L1, RNP: ribonucleoprotein, IFN: interferon, aCL: anticardiolipin, GFAP: glial fibrillary acid protein, APRIL: a proliferation-inducing protein, IP: interferon-gamma induced protein, CCL: chemokine ligand, PAI: plasminogen activator inhibitor, LAC: lupus anticoagulant.

**Figure 2:**
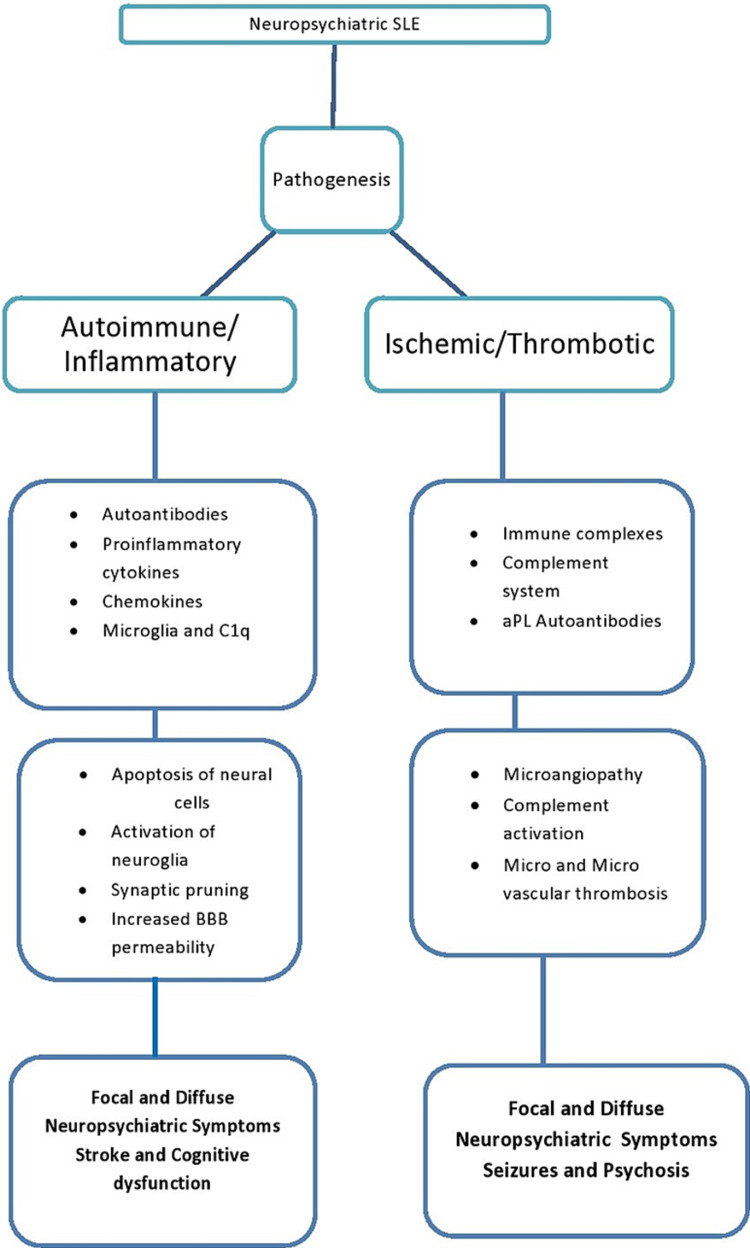
Pathogenic pathways for neuropsychiatric SLE BBB: Blood-brain barrier, aPL: Antiphospholipid autoantibodies.

Pathologic mechanisms in SLE include loss of immune tolerance to cellular nuclear antigen, production of autoantibodies, and deposition of immune complexes leading to complement activation, tissue inflammation, and cellular apoptosis. Altered B and T-cell activation, anomalous apoptotic material clearance, and activation of type I interferon (IFN) are prominent features involved in pathogenesis [20]. Pathophysiological mechanisms in NPSLE remain poorly understood; however, several risk factors have been proposed as a potential culprit in the pathogenesis of NPSLE (Figure 1) [6,21]. Moreover, two pathologic mechanisms have been proposed contributing to NPSLE: (1) Autoimmune or inflammatory pathway leading to NP manifestations due to inflammatory mediators or autoantibodies with either intrathecal immune complex formation or disrupted blood-brain barrier (BBB), and (2) ischemic or thrombotic pathway leading to cerebral microangiopathy, vascular occlusion, and hemorrhage. Accelerated atherosclerosis, immune complex deposition, and immune-mediated vascular injury interplay in this pathway (Figure 2) [22,23]. In the majority of the cases, both pathologic mechanisms coexist and are responsible for manifesting a broad spectrum of NP signs and symptoms. Primary NPSLE involves both inflammatory and ischemic NPLSE, and secondary NPSLE includes those patients who have NP manifestations due to SLE-related organ damage or SLE medications.

Autoantibodies are considered a potential culprit in the pathogenesis of NPSLE. Significant mediators include anti-endothelial, anti-ribosomal P, anti-neuronal antibodies. Anti-ribosomal ant anti-NR2 antibodies induce neuronal cell death when passing through the disrupted BBB. The presence of anti-NR2 antibodies in cerebrospinal fluid (CSF) results in motor function impairment and disturbance in visuospatial processing [24,25]. Other autoantibodies and their association with NPSLE are under analysis (Table 2) [26-29]. There is a dire need to develop and confirm an array of specific and sensitive laboratory serum or CSF biomarkers, radiological imaging changes for reliable apprehension of all different aspects of NPSLE heterogeneousness and further improve prevention, diagnosis, and management [30].

**Table 2:** Potentially relevant autoantibodies in neuropsychiatric SLE GAPDH: Glyceraldehyde 3-phosphate dehydrogenase, ab: antibody, SBSN: supra-basin, CSF, cerebrospinal fluid, BC RNA: brain cytoplasmic ribonucleic acid, CNS: central nervous system.

Autoantibody	Induction	Clinical finding
Anti-GAPDH Ab [26]	Induce neurite interaction and impairment of neuronal plasticity by blocking and binding of synaptic molecules.	Antibody level increases in SLE and NPSLE and is associated with generalized disease activity, cognitive dysfunction, and psychiatric manifestations.
Anti-SBSN Ab [27]	Astrocytes exposed to antibodies have altered senescence and autophagy pathways.	It can be a helpful marker to differential NPSLE from SLE in the absence of NP symptoms because anti-SBSN antibody and the associated immune complex were only detected in the CSF of NPSLE.
Anti-UCH-L1 Ab [28]	Detected in the CSF of NPSLE patients and has been prosed as a potential biomarker of NPSLE.	NPSLE patients had significantly increased levels of CSF anti-UCH-L1. In addition, this marker was associated with enhanced disease severity and generalized disease activity.
Anti-BC RNA [29]	Responsible for diminished delivery of BC RNA to synaptodendritic sites in the brain.	Lack of BC RNA in CNS causes phenotypic abnormalities and cognitive decline.

Diagnostic approach and challenges

There are no specific criteria to diagnose NPSLE and are based on the diagnosis of exclusion and expert opinion. In all the patients presented with inexplicable neuropsychiatric symptoms or manifestations suggestive of NP disease, the primary step would be to investigate thoroughly and categorize the NP manifestations and exclude other common causes such as metabolic abnormalities, infections, or drug abuse [31]. Thorough clinical assessment involves neurological and psychiatric evaluation. Further evaluation includes assessing general SLE activity, cardiovascular risk factors, atherosclerotic disease, and thrombotic events. NPSLE diagnosis is achieved using different clinical, serological, immunological, electrophysiological, and neuroimaging studies [13]. Magro-Checa et al. proposed a diagnostic approach in NPSLE patients based on the clinical presentation of SLE patients (Table 3) [32].

**Table 3:** Diagnostic approach in neuropsychiatric SLE LP: lumbar puncture, MRI: magnetic resonance imaging, EMG: electromyography, NCSL nerve conduction study, MS: multiple sclerosis, DM: diabetes mellitus, AchR: acetylcholine receptor, MRA: magnetic resonance arteriography, EEG: electroencephalography, AVM: arteriovenous malformation, MuSK: muscle-specific tyrosine kinase, TIA: transient ischemic attack, LETM: longitudinally extensive transverse myelitis, EKG: echocardiography, GBS: Guillain Barre syndrome, CVA: cerebrovascular accident

Clinical scenario	Proposed workup	Possible indications
Seizures, Acute confusional state	LP, MRI	Exclude infection/malignancy/neurological disease
	EEG	For seizure confirmation
	Additional testing	Serologic testing for infectious workup/microbial cultures
Autonomic disorder, Polyneuropathy	Autonomic testing	Assessment of severity and parts involved
	NCS, EMG	Neuropathy characterization
	Additional testing	Exclude DM/uremic neuropathy/vitamin deficiencies/celiac disease/AchR/exclude infections
Myasthenia gravis, Cranial neuropathy	MRI	Exclude MS/compression of cranial nerves
	CT	Exclude thyroid disease
	Additional testing	Exclude thyroid disease, look for specific antibodies (AchR, MuSK, LRP4)
Aseptic meningitis	LP, MRI	Exclude infection/subarachnoid hemorrhage
	Additional testing and NSAID use	Infectious workup/cultures
Cerebrovascular disease (TIA/Stroke)	MRI	Exclude infarcts
	EKG	Excludes cardiac causes (thromboembolic) of CVA
	Doppler ultrasonography of carotid arteries	Exclude cardiac (thromboembolic) causes of CVA
	MRA/LP	Exclude cerebral vasculitis
Myelopathy, Plexopathy, Movement disorder	MRI brain	Exclude infection/MS/optic neuritis/malignancy
	MRI spine	Exclude infection/malignancy/AVM, confirm LETM
	LP	Exclude infection/oligoclonal bands
	Additional testing	Workup for infectious disease/Wilson disease
Psychosis, Cognitive dysfunction	MRI	Exclude infection/infarction/malignancy
	Additional testing	Exclude DM/thyroid disease/vitamin deficiencies/pheochromocytoma
GBS	MRI spine	Exclude myelopathies
	Additional testing	Infectious workup

NPSLE may be the sole or primary presentation of SLE and frequently manifests when SLE is clinically and serologically active. Therefore, by combining the clinical evaluation, serological studies, and imaging results, we can proclaim that patient has active NPSLE or having symptoms due to other causes [5]. Zhang et al. predicted the specificity and sensitivity of clinical manifestations in NPSLE 37.1% and 97.4% with a positive predictive value of 70%. Moreover, they highlighted the sensitivity and specificity of positive antibody tests in NPSLE 84% and 53%, respectively, with a positive predictive value of 71.1%. He also reported that the SLE disease activity index score and positive skin manifestation might be helpful in the diagnosis of NPSLE [14].

Despite extensive clinical research, none of the laboratory and neuroimaging biomarkers have been proven accurate or reliable using clinical practice to diagnose NPSLE. So, there is an unmet need for diagnostic biomarkers in serum and CSF and innovation in imaging modalities to determine the ascription of NP manifestations to SLE. Although some autoantibodies have been suggested as a potential biomarker, only a few antibodies such as antineuronal, anti-ribosomal P, and ant-NR2 antibodies have met the exploratory criteria and are being used in the diagnosis and therapeutic decisions [24,25]. Among cytokines, elevated interleukin-6 level in the CSF has shown a positive correlation with diffuse NPSLE, such as an acute confusional state [20,33]. Identification of more specific neural antigens and a better understanding of BBB are warranted from the mounting contributions of genomics and immunoproteomics [34]. Magnetic resonance imaging (MRI) of the brain is considered a gold standard for assessing NPSLE patients. However, there is still a clinical and radiological paradox. More than 50% of the patients with a clinical diagnosis of NPSLE have no obvious abnormality on MRI and vice versa [33]. Thus, a comprehensive approach and imaging studies are warranted to overcome this confusion. In addition, there is a possibility of improving the attribution of NP manifestations to SLE and non-SLE causes and advancing impartial neuroimaging outcomes to observe the new therapy response in NPSLE patients [35].

Management and challenges

NPSLE management is challenging based on obscure signs and symptoms for diagnosis, attributing these manifestations to SLE and the presence of limited or lack of management armamentarium. Therefore, management of NPSLE is focused on symptomatic treatment such as antiepileptic for seizures, anxiolytics or antipsychotics for psychiatric manifestations, antihypertensive drugs for hypertension, and correction of metabolic derangements [31]. In addition, the underlying SLE process of neuropsychiatric manifestations should also be halted, such as thromboembolic disorder and inflammation-driven syndrome [32]. The European League Against Rheumatism (EULAR) published a consensus and gave possible recommendations for NPSLE management. These recommendations include a general therapeutic approach that does not vary from non-SLE patients presenting with neuropsychiatric manifestations. Conventional treatment includes symptomatic management, nonpharmacological interventions, and corrections of underlying and aggravating factors [31,32].

First, non-SLE factors should be managed appropriately using non-SLE-specific interventions. A study reported the beneficial effects of psychotherapy in managing anxiety, depression and improving quality of life [36]. Antidepressants and anxiolytics are also often used, and their use reported positive outcomes in improving cognitive functions in SLE patients with anxiety and depression; however, their use in mood disorders is variable [37]. The use of antiepileptics for seizures in SLE has shown favorable efficacy, and antipsychotics are used for SLE psychosis [38,39]. Cognitive dysfunction in SLE is managed with a meta context behavioral rehabilitation strategy. A non-randomized study on rehabilitation strategy highlighted a 100% retention rate with memory self-efficacy and improved quality of life [40]. There is an unmet need for a controlled study to delineate the pharmacotherapy components of this intervention.

**Figure 3:**
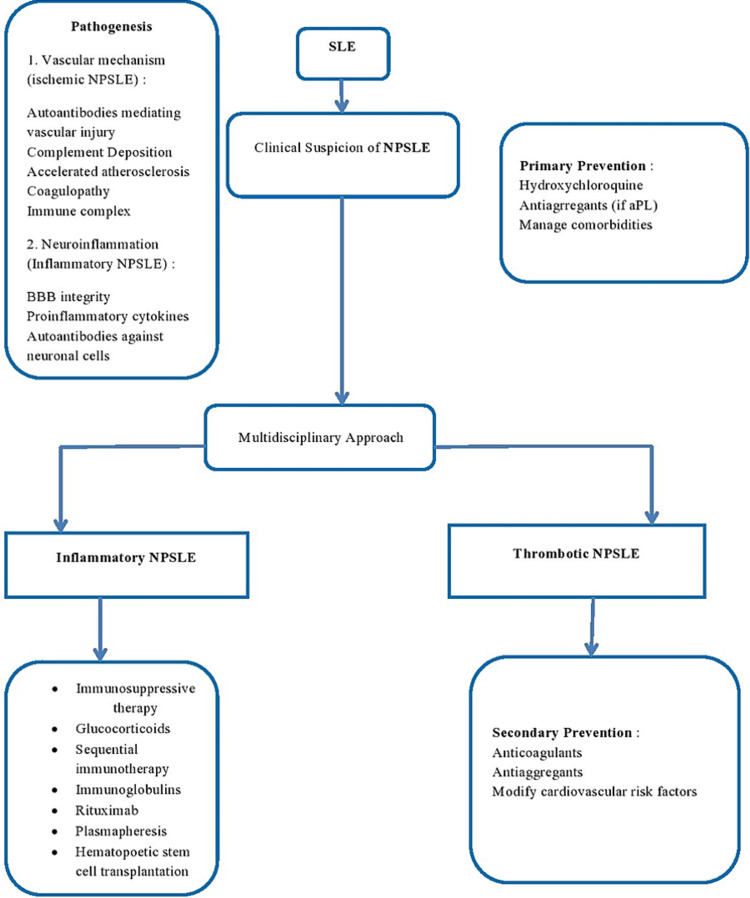
Treatment options in neuropsychiatric SLE BBB: Blood-brain barrier, NPSLE: Neuropsychiatric systemic lupus erythematosus, aPL: Antiphospholipid autoantibodies.

Some therapies in NPSLE are empirical due to the lack of controlled clinical trials. Pharmacological treatment is directed to treat inflammation or prevent thrombotic events in clinical practice, depending on the alleged underlying pathophysiology [32]. In patients with manifestations of generalized lupus activity or immune-mediated injury, the commencement of immunosuppressants such as corticosteroids is warranted alone or in combination with other immunosuppressive therapy, including azathioprine, cyclophosphamide, and mycophenolate mofetil. The main objective of immunotherapy is to resolve or stabilize the symptoms [31]. Intravenous cyclophosphamide and oral prednisolone are the only agents tested in NPSLE with positive results [41]. Patients taking antimalarial drugs have reported a lower risk of seizures [38]. Other adjunctive therapies include statins in patients with arterial or recurrent venous thrombosis and nonsteroidal anti-inflammatory drugs (NSAID) for pain [42]. However, NSAID use in SLE is associated with an increased incidence of recurrent aseptic meningitis [43]. Ischemic NPSLE is managed with anticoagulation and antiplatelet therapy, particularly if the patients have positive antiphospholipid antibodies [39]. In most patients, inflammatory and ischemic NPSLE coexist; authors suggest using a combination of therapies, including immunosuppressive, anticoagulation, and antiplatelet therapy [35]. Although patients with NPSLE are individually tailored, a treatment algorithm based on current evidence is shown in Figure 3 [31].

There are limited data related to the efficacy of biologic agents in NPSLE. Using rituximab alone or in combination with conventional immunosuppressants, including cyclophosphamide, has reported favorable outcomes in open studies of B-lymphocyte depletion but requires even more precise data [44]. Clinical use of belimumab in NPSLE has shown efficacy only in patients with headaches, not in those who had significant NPSLE events [45]. A recent phase III clinical trial reported the positive result of anifrolumab in NPSLE; however, this trial excluded patients with severe disease, and further results are yet to be reported [46]. Thus, there is an unmet need for novel therapies targeting the BBB (disruption results in exposure of autoantibodies to the brain), cytokines (IL-6, type I IFN), and microglial cells for NPSLE management. In addition, new clinical trials should evaluate the potential options such as neuroimaging, validating outcome measures, and therapy for non-emergent neuropsychiatric events, including mood disorders [36,37].

## Conclusions

New therapies and targets are being established with a better understanding of immune mechanisms involved in active SLE. However, there is no potential agent against NPSLE. Therefore, evaluating and designing effective interventions requires an understanding of the pathophysiology that led to NPSLE. In addition, many clinical situations need to be further explained. Currently, the mechanisms underlying the NPSLE remain poorly understood. We lack in vivo imaging biomarker that provides direct evidence of BBB dysfunction restricting the diagnosis and treatment for patients with NPSLE. Furthermore, there are limited controlled clinical trials for evaluating NPSLE treatment, and current management guidelines are based on expert recommendations and small observational cohort studies. It is challenging to run such trials due to the sheer number of patients and collaboration between centers from different countries. This review summarizes recent insights regarding NPSLE that may serve as a basis for future advances. Further research should be directed to identify biomarkers for NPSLE and develop clinical trials for novel and established drugs in SLE patients with NP manifestations. In addition, a multidisciplinary effort involving rheumatologists, neurologists, and psychologists should be involved in both diagnosing and treating NPSLE in clinical settings, and the rheumatologists should take the lead on these initiatives.
